# Effect of the Amount and Particle Size of Wheat Fiber on the Physicochemical Properties and Gel Morphology of Starches

**DOI:** 10.1371/journal.pone.0128665

**Published:** 2015-06-08

**Authors:** Qingjie Sun, Min Wu, Xianghui Bu, Liu Xiong

**Affiliations:** College of Food Science and Engineering, Qingdao Agricultural University, Qingdao, China; USDA-ARS, UNITED STATES

## Abstract

Effects of added wheat fiber, with different levels and particle sizes, on the physicochemical properties and gel morphology of wheat starch and mung bean starch were investigated, using rapid visco analyzer (RVA), texture analyzer (TPA) and scanning electron microscopy (SEM). Each starch was added with wheat fiber at 10, 20, 30 and 40% (weight basis, g/100g), and different sizes of 60, 100 and 180 mesh, respectively. The peak viscosity (PV) of starches with wheat fiber were higher than the control. Starches had the highest PV with 40%, 60 mesh wheat fiber. The starches with wheat fiber showed higher hardness when compared to the control. Wheat starch and mung bean starch, with 40%, 60 mesh wheat fiber, had the highest hardnesses of 147.78 and 1032.11g, respectively. SEM showed that the dense honeycomb structure of starch gel was diminished with increasing wheat fiber. Additionally, the number of internal pores was reduced, and a large lamellar structure was formed.

## Introduction

Starch is one of the most available natural ingredients for the food industry, either as a main raw material or as a food additive. As a low cost agricultural commodity, derived primarily from cereal and tuber crops, starch is widely used in a variety of food and beverage products [1]. Mung bean starch is the best raw material to produce high quality starch noodles, vermicelli and sheet jelly, because of its high amylose content, restricted swelling during gelatinization, and the high shear resistance of its paste [2]. Wheat starch, from refined grains, is mainly used to make traditional Asian foods and baked products [3]. In the food industry, wheat starch is also used as a food thickener, gelling agent or stabilizer [4]. Blends of native starches and other polysaccharide hydrocolloids have been used in the modern food industry to modify and control the texture and eating quality of food products [5].

Dietary fiber (DF) is comprised of a wide array of carbohydrate-based non-digestible food components, with great physiological significance. DF has attracted increasing interest in recent years, as many studies have revealed that it may be involved in disease preventing and health promoting activities, including the attenuation of blood cholesterol and/or glucose, a laxative effect, and a reduction in the risk of colon cancer, heart disease and obesity [6,7]. Wheat fiber is used as the dietary fiber supplement in the food industry. The carbohydrate moiety of DF includes cellulose, β-glucans and hemicelluloses, such as arabinoxylans and arabinogalactans, pectins, gums and mucilages [8]. So there is a growing emphasis on the development of high dietary fiber foods.

When natural fibers are added to starches, an improvement in the mechanical properties and performance of the composite is observed [9,10]. Different fibers, such as that from cotton, pea, coir, vegetal and barley-glucan, when added to starches the resulting hydrophilicity, pasting characteristics, tensile and thermal properties of the composite have been studied [11,12,13]. Fiber-rich foods can play an important role in human health and disease prevention. Adding fiber to starch foods will become the focus of the research and market development. To the best of our knowledge, the effects of wheat fiber, with different levels and particle sizes, on the physicochemical properties and gel morphology of wheat starch and mung bean starch have not been studied. Our studies will add to the understanding of these topics and could provide some theoretical bases for the development and utilization of starch products with enriched wheat fiber.

## Materials and Methods

### 2.1 Materials

Wheat starch (amylose content 28%) and mung bean starch (amylose content 40%) were supplied by National Starch and Chemical Limited in Shanghai, China. Wheat fiber (dry basis) was purchased from Shanghai NuoShen Food Trading, Co., Ltd, Shanghai, China. The purity of wheat fiber was 99.99%.

### 2.2 Wheat fiber preparation

The wheat fibers were pulverized using a grinder and sieved using 60, 100 and 180 mesh (the pore sizes are 0.3mm, 0.15mm and 0.09mm, respectively) sieves, in order to obtain three grades of wheat fibers.

### 2.3 Determination of water holding capacity

Water holding capacity of wheat fiber was measured according to the approach of Fabrizio Esposito [14]. Wheat fibers (2.500g) with different sizes of 180 mesh, 100 mesh and 60 mesh were put in three centrifuge tubes and 30 mL water was added, respectively. The mixtures were kept for 0.5h at 25°C. After centrifugation at 1428 g for 10 min, the supernatant was discarded. The quality of residues remained in the centrifuge tubes were weighed. The water holding capacity of wheat fiber was calculated according to the following formula (g/g).

Water holding capacity = (Dietary fiber wet weight - Dietary fiber dry weight) / Dietary fiber dry weight

### 2.4 Determination of pasting properties

The pasting properties of wheat starch and mung bean starch with wheat fiber added were evaluated using a Rapid Visco Analyzer (RVA-4, Newport Scientific, Warriewood, Australia), as described by Singh et al. [15]. Starch (3g, moisture content 14%), added extra different particle sizes (60, 100 and 180 mesh) of wheat fiber (40 wt.% on a starch basis), and different added amounts of wheat fiber (60 mesh, 10, 20, 30 and 40 wt.% on a starch basis), was directly weighed in the RVA canister, and deionized water was added to obtain a total sample weight of 28 g. There were two independent sampling, each of which was measured in triplicate. There were a total of 9 data points in three groups. The means of three groups from repeated measurements are then averaged and evaluated with a SD. These calculations are done by spss software.

A programmed heating and cooling cycle was used, where the samples were held at 50°C for 1 min, heated to 95°C in 3.7 min (heating and cooling rate 12°C/min), held at 95°C for 2.5 min, before cooling to 50°C in 3.8 min, and finally held at 50°C for 2 min. The parameters that were recorded included pasting temperature (PT); peak viscosity (PV); hot paste viscosity (HPV) (minimum viscosity at 95°C); cool paste viscosity (CPV) (final viscosity at 50°C); breakdown (BD) (= PV–HPV); and set back (SB) (= CPV–HPV). All measurements were replicated three times.

### 2.5 Determination of textural properties

The gelatinized mixture in the canister, after the RVA measurement, was kept overnight at 4°C to form a solid gel. The texture of the gel was then determined using a texture analyzer (TA-XT2, Texture Technologies Corp, US). The gel with a dimension of 37 mm in diameter and 20 mm in height was compressed at a speed of 1.0 mm/s, to a distance of 10.0 mm, with a stainless steel punch probe 5.0 mm in diameter. And all measurements were replicated three times [16].

### 2.6 Scanning electron microscopy (SEM)

The pastes of wheat starch with different amounts and different particle sizes of wheat fiber, were quickly put into an ultra-low temperature freezer (-70°C). The microstructure of the samples after freeze drying for 48 h was observed using SEM. The surface topography of the samples was observed with SEM, using the method of Kim et al. [17]. A dry, finely ground sample was placed on double-sided Scotch tape, mounted on an aluminum specimen holder, and coated with a thin film of gold under vacuum conditions. The samples were observed using a Jeol scanning electron microscope (JSM 840, Jeol, Japan).

### 2.7 Statistical analysis

All the experiments were conducted at least twice, each of which was measured in triplicate. The means of two groups from triplicate measurements were then averaged and evaluated with a SD. Experimental data groups were analyzed using the analysis of variance (ANOVA), and expressed as mean values ± standard deviations. Differences were considered at a significance level of 95% (p<0.05), The Pearson’s correlation coefficients of the parameters were calculated using SPSS 17.0 (SPSS, Chicago, IL).

## Results and Discussion

### 3.1 Determination of pasting properties

The paste viscogram data of the wheat starch and mung bean starch, with different amounts of wheat fiber, are shown in Table 1. When the starch granules were heated in excess water, they absorbed the water and swelled to 10–20 times their original size. As a consequence of this swelling, the granules burst open and amylose leached out. This resulted in an increase in the viscosity of the suspension, and later, a continuous gel phase was formed by the amylose. In this continuous phase, amylopectin and swollen starch granules were embedded [18].

**Table 1:** Effect of the amount of wheat fiber on pasting properties of wheat starch and mung bean starch.

Sample*	Pasting temperature (°C)	Peak viscosity (RVU)	Hot paste viscosity (RVU)	Cool paste viscosity (RVU)	Breakdown (RVU)	Set back (RVU)
WS	89.5±0.05^d^	233.58±0.03^a^	164.08±0.03^a^	279.17±1.23^a^	69.50±0.1^a^	115.08±1.01^a^
WSW-10	84.2±0.08^c^	277.08±0.15^b^	203.92±0.31^b^	318.50±0.12^b^	73.17±0.0^a^	114.58±0.08^a^
WSW-20	74.7±0.06^b^	322.42±0.08^c^	236.08±0.05^c^	358.17±0.11^c^	86.33±0.1^b^	122.08±0.06^a^
WSW-30	69.4±0.11^a^	368.00±0.22^d^	251.25±1.10^d^	388.75±0.23^d^	116.75±0.9^c^	137.50±0.10^b^
WSW-40	68.1±0.12^a^	470.67±0.09^e^	247.25±3.12^d^	409.33±0.18^e^	223.42±0.45^d^	162.08±0.06^c^
MS	73.6±1.12^b^	306.75±7.88^a^	197.92±6.11^a^	369.58±4.24^a^	108.83±6.12^a^	171.67±6.02^a^
MSW-10	73.2±0.03^b^	345.25±3.15^b^	202.08±2.09^a^	385.25±1.13^b^	143.17±0.11^b^	183.17±5.32^b^
MSW-20	73.0±0.06^b^	423.00±1.22^c^	247.58±3.03^b^	413.50±2.22^c^	175.42±0.08^c^	205.92±3.11^c^
MSW-30	71.8±0.03^a^	477.42±2.13^d^	243.50±2.09^b^	439.75±0.27^d^	233.92±0.11^d^	196.25±0.02^d^
MSW-40	71.8±0.06^a^	554.67±0.22^e^	273.17±0.11^c^	530.83±1.01^e^	281.50±0.04^e^	257.67±0.15^e^

All data represent the mean of two replicates. Mean ± standard deviation. Different letters in the same column are significantly different (p < 0.05).

* WS, WSW-10, WSW-20, WSW-30, WSW-40 mean wheat starch and that with 10, 20, 30, and 40% wheat fiber, respectively; MS, MSW-10, MSW-20, MSW-30, MSW-40 mean mung bean starch and that with 10, 20, 30, and 40% wheat fiber, respectively.

Compared with the control, the PT of the wheat starch and mung bean starch, with 40% wheat fiber, lessened from 89.5 and 73.6°C to 68.1 and 71.8°C, respectively. The PT represented the temperature when the starch slurry began to generate viscosity. One possible reason was more water absorbed by the fiber, which led to a relative increase in the concentration of the starches. Thus, the viscosity would generate at a lower temperature, and the PT was lessened with increasing wheat fiber. This was similar to the results of Tao Feng et al. [19]. When wheat fiber was added, the PT lessened around 20°C for wheat starch, and just 2°C for mung bean starch, this maybe due to mung bean starch containing more amylose than wheat starch, thus had the stronger capability to resist the effects of wheat fiber.

With increasing wheat fiber, the PV of the two starches increased from 233.58 and 306.75 RVU, to 470.67 and 554.67 RVU, respectively. These values were approximately two times larger than those of the control. There were two possible reasons for this: on the one hand, the wheat fiber had a higher water-holding capacity, contributing to part of the viscosity in the heating process, and absorbing more water than the wheat starch and mung bean starch. After adding the wheat fiber, the starch’s capability of absorbing water was reduced, so the concentrations of the starches increased, resulting in an increase in the PV. On the other hand, the starch and wheat fiber particles promoted interactions between them. These integrated factors led to the increase in viscosity. These results were similar to the results of Mohammed S. Alamri et al., Phoency Lai et al. and Ravi Sharma et al. [20, 21, 22].

The HPV, CPV, BD and SB of the wheat starch and mung bean starch added to the wheat fiber were also increased, compared with those of the control. The increase in HPV, CPV and BD were related to the increase in PV. The SB represented the retrogradation phenomenon of the starch paste. The increase in the SB may be due to the high water holding capability of the wheat fiber, which caused the concentration of the amylose to increase, thus contributing to the retrogradation of the starch paste.

The paste viscogram data of the wheat starch and mung bean starch with different particle sizes of wheat fiber are shown in Table 2. With the increasing particle size of 40% added wheat fiber, the PVs of both starches increased, while the PTs showed no significant changes. Compared to the control, the PVs of the wheat starch and mung bean starch, with the 40%, 60 mesh wheat fiber, increased from 233.58 and 306.75 RVU, to 449.42 and 540.58 RVU, respectively. These values were about two times larger than those of the control.

**Table 2:** Effect of the particle size of wheat fiber on pasting properties of wheat starch and mung bean starch.

Sample*	Pasting temperature (°C)	Peak viscosity (RVU)	Hot paste viscosity (RVU)	Cool paste viscosity (RVU)	Breakdown (RVU)	Set back (RVU)
WS	89.5±0.05^c^	233.58±0.03^a^	164.08±0.03^b^	279.17±1.23^a^	69.50±0.12^a^	115.08±1.01^a^
WSW_180_	68.0±0.24^b^	370.83±1.14^b^	157.33±3.01^a^	286.83±1.04^b^	213.50±3.01^b^	129.50±2.61^a^
WSW_100_	66.7±0.06^a^	424.33±3.32^c^	184.25±2.33^c^	330.08±2.22^c^	240.08±0.23^c^	145.83±1.03^b^
WSW_60_	69.6±0.12^b^	449.42±2.23^d^	209.00±1.12^d^	363.17±1.18^d^	240.42±0.12^c^	154.17±0.12^c^
MS	73.6±1.12^b^	306.75±7.88^a^	197.92±6.11^a^	369.58±4.24^a^	108.83±6.12^a^	171.67±6.02^a^
MSW_180_	72.6±0.12^b^	471.92±5.12^b^	272.00±7.16^b^	546.92±0.03^d^	199.08±6.03^b^	274.50±9.12^b^
MSW_100_	71.6±0.06^a^	537.33±4.34^c^	253.33±8.15^b^	507.46±0.07^b^	280.83±7.45^c^	254.92±7.23^b^
MSW_60_	71.4±0.02^a^	540.58±6.43^c^	271.83±9.05^b^	540.00±0.12^c^	268.75±0.03^c^	269.42±8.22^b^

All data represent the mean of two replicates. Mean ± standard deviation. Different letters in the same column are significantly different (p < 0.05).

* WS, WSW_180_, WSW_100_, WSW_60_ mean wheat starch and that with 180-mesh, 100-mesh, 60-mesh wheat fiber, respectively; MS, MSW_180_, MSW_100_, MSW_60_ mean mung bean starch and that with 180-mesh, 100-mesh, 60-mesh wheat fiber, respectively.

The PVs of the starches with large particle sizes of wheat fiber increased more significantly. The water holding capacity of 180, 100, 60 mesh wheat fiber were 7.0, 7.6, 8.1 g/g, respectively. So one possible reason was that the large particles had been broken down to a slight degree, compared with the small particles. The organization and structure of the fiber were well maintained, so the water holding capacity of the large particles were stronger. The larger wheat fiber (60 mesh) could absorb much more water during the heating process, therefore, the water absorbed by the starches was reduced and the PV increased. These studies were similar to the results of James N. BeMiller et al. and Chien-Chun Huang et al. [23, 24].

Compared with the control, the HPV, CPV, BD and SB of the wheat starch and mung bean starch, with different particle sizes of wheat fiber, were increased. The increase in the SB may be due to the interaction that occurred between the different particle sizes of wheat fiber, and the leached amylose from the swollen starch granules. The 180 mesh wheat fiber had been ground down even further than the 60 mesh, and the large particle size wheat fiber had a stronger effect on the SB of the starch paste.

### 3.2 Determination of textural properties

The effects of the amount of wheat fiber on the gel texture properties of the wheat starch and mung bean starch are shown in Table 3. The hardness and chewiness of the wheat starch paste and mung bean starch paste increased with increasing wheat fiber, the cohesiveness lessened, and the springiness showed no significant changes (Table 3).

**Table 3:** Effect of the amount of wheat fiber on gel texture properties of wheat starch and mung bean starch.

Sample*	Hardness(g)	Springiness	Cohesiveness	Gumminess	Chewiness
WS	61.82±0.05^a^	0.96±0.01^d^	0.95±0.01^d^	58.72±0.05^a^	56.38±0.02^a^
WSW-10	90.66±0.12^b^	0.95±0.03^c^	0.84±0.03^c^	76.15±0.02^b^	72.35±0.01^b^
WSW-20	100.49±0.06^c^	0.95±0.01^c^	0.86±0.04^c^	86.42±0.13^d^	82.10±0.14^d^
WSW-30	125.76±0.16^d^	0.94±0.02^b^	0.65±0.03^b^	81.74±0.02^c^	76.84±0.05^c^
WSW-40	147.78±0.12^e^	0.93±0.02^a^	0.52±0.01^a^	76.85±0.12^b^	71.47±0.12^b^
MS	530.52±0.12^a^	0.99±0.01^d^	0.65±0.01^e^	344.83±0.03^a^	341.38±0.12^a^
MSW-10	820.26±1.13^b^	0.98±0.02^c^	0.59±0.01^d^	483.95±0.08^b^	474.27±0.08^c^
MSW-20	857.59±4.02^c^	0.97±0.01^b^	0.57±0.02^c^	488.83±0.10^c^	474.16±0.12^c^
MSW-30	1015.31±2.07^d^	0.92±0.02^a^	0.49±0.05^b^	497.50±0.32^d^	457.70±0.02^b^
MSW-40	1032.11±1.03^e^	0.99±0.01^d^	0.47±0.01^a^	485.09±1.03^b^	480.24±1.05^d^

All data represent the mean of two replicates. Mean ± standard deviation. Different letters in the same column are significantly different (p < 0.05).

* WS, WSW-10, WSW-20, WSW-30, WSW-40 mean wheat starch and that with 10%, 20%, 30%, 40% wheat fiber, respectively; MS, MSW-10, MSW-20, MSW-30, MSW-40 mean mung bean starch and that with 10%, 20%, 30%, 40% wheat fiber, respectively.

Compared with the control, the hardness of the two starches with 40% wheat fiber increased about 2 times, from 61.82 and 530.52 g, to 147.78 and 1032.11 g, respectively. The hardness represented the integrity of the starch gel. The formation of hardness was due to the gelatinized starch experiencing a period of cooling with a rearrangement occurring among the starch molecules. The amylose retrogradation occurred forming the starch gel. Wheat fiber and starches may interact with each other, causing a large number of the elastic active chains to entangle into a gelling network [25]. In fact, when natural fibers were used as reinforcements in the starches, an obvious improvement in the mechanical properties and performance of the composite was expected. This was due to both fibers and starches were the polysaccharide polymers composed of D-glucopyranose units linked by 1,4 glycosidic bond, and contained a large number of hydroxyl groups. particularly in the cellulose chains [26]. All of these relationships are based largely on the intrinsic structure and properties of the wheat fiber.

Fiber is a high molecular weight linear homopolymer, composed of repeating β-D-glucopyranosyl units joined by 1, 4-glycosidic bonds [21]. As an insoluble material, wheat fiber has a stronger rigid structure than the starch gel formed by pasting. The fiber-starch mixed system forms a stronger structure than the single starch system. The added wheat fiber could interact with amylose and amylopectin in the starch gel, and increased the hardness of the starch gel.

The effects of the particle size of the wheat fiber on the gel texture properties of the wheat starch and mung bean starch are shown in Table 4. With the increase in particle size of the 40% wheat fiber in the wheat starch and mung bean starch, the hardness and chewiness of the starch paste increased and the cohesiveness lessened, while the springiness did not change significantly (Table 4).

**Table 4:** Effect of the particle size of wheat fiber on gel texture properties of wheat starch and mung bean starch.

Sample^a^	Hardness(g)	Springiness	Cohesiveness	Gumminess	Chewiness
WS	61.82±0.05^a^	0.96±0.01^c^	0.95±0.01^c^	58.72±0.05^a^	56.38±0.02^c^
WSW_180_	120.28±0.34^b^	0.87±0.06^a^	0.49±0.03^a^	58.93±1.12^a^	51.27±0.16^a^
WSW_100_	126.20±1.01^c^	0.86±0.03^a^	0.49±0.02^a^	61.84±0.30^b^	53.18±0.32^b^
WSW_60_	147.78±0.12^d^	0.93±0.02^b^	0.52±0.01^b^	76.85±0.12^c^	71.47±0.12^d^
MS	530.52±0.12^a^	0.99±0.01^a^	0.65±0.01^c^	344.83±0.03^a^	341.38±0.12^a^
MSW_180_	870.26±3.01^b^	0.98±0.01^a^	0.46±0.00^a^	400.31±3.43^b^	392.31±2.05^b^
MSW_100_	980.03±2.44^c^	0.98±0.00^a^	0.46±0.01^a^	450.82±0.87^c^	441.80±0.54^c^
MSW_60_	1032.11±1.03^d^	0.99±0.01^a^	0.47±0.01^b^	485.09±1.03^d^	480.24±1.05^d^

All data represent the mean of two replicates. Mean ± standard deviation. Different letters in the same column are significantly different (p < 0.05).

* WS, WSW_180_, WSW_100_, WSW_60_ mean wheat starch and that with 180-mesh, 100-mesh, 60-mesh wheat fiber, respectively; MS, MSW_180_, MSW_100_, MSW_60_ mean mung bean starch and that with 180-mesh, 100-mesh, 60-mesh wheat fiber, respectively.

The hardness of the two starches with 60 mesh wheat fiber increased about two times, from 61.82 and 530.52 g, to 147.78 and 1032.11 g, when compared with the control. The large particle size wheat fiber made a more significant impact on the hardness of the fiber-starch mixtures than the small particles. During the pasting process, the extent of the granule swelling and disintegration, as well as the release of amylose, depended on the type of starch and its concentration, temperature, presence of other solutes, and the shear or agitation applied during heating. As the gelatinized dispersion of starch was cooled, a loose paste or gel would form, depending on the concentration [27]. In the mixture system of starch and wheat fiber, the wheat fiber was structurally stronger than the starch. Meanwhile, the larger particle size wheat fiber could maintain a stronger rigid structure, compared with the small particle size wheat fiber. The physical and chemical properties of the 60 mesh wheat fiber were better than that of the 180 mesh. The large particles had a greater advantage in competing for the water, and formed a strong network structure. In a similar way, vegetal fiber and flax fiber were blended with cassava starch and pea starch to enhance the mechanical properties [11,28].

### 3.3 Determination of gel morphology

**Fig 1:**
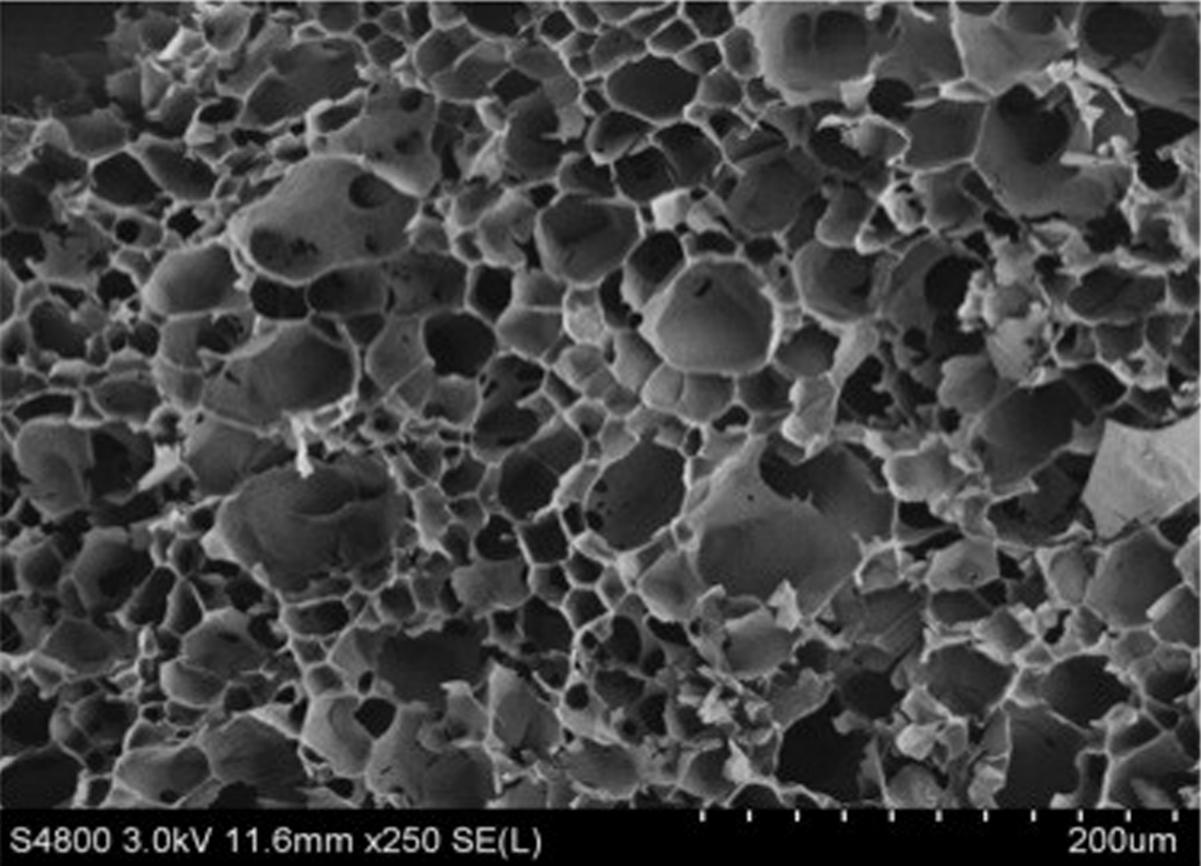
Scanning electron micrographs (SEM) of gelatinized wheat starch gel.

**Fig 2:**
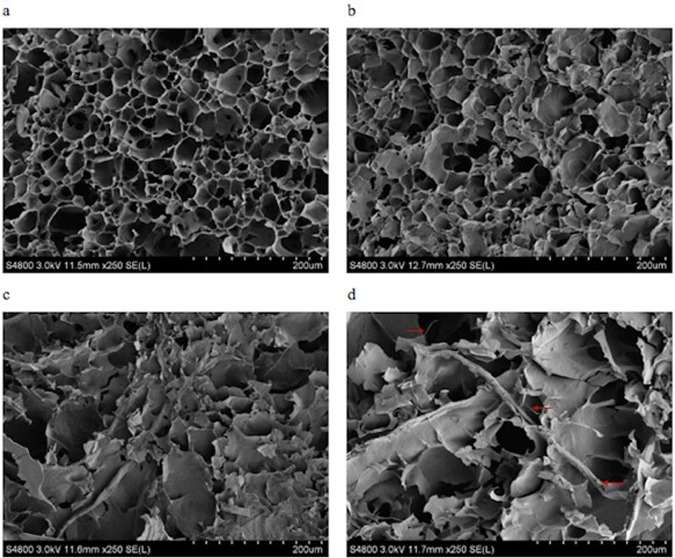
Scanning electron micrographs (SEM) of gelatinized wheat starch gel with 10, 20, 30, 40% 60-mesh wheat fiber.

**Fig 3:**
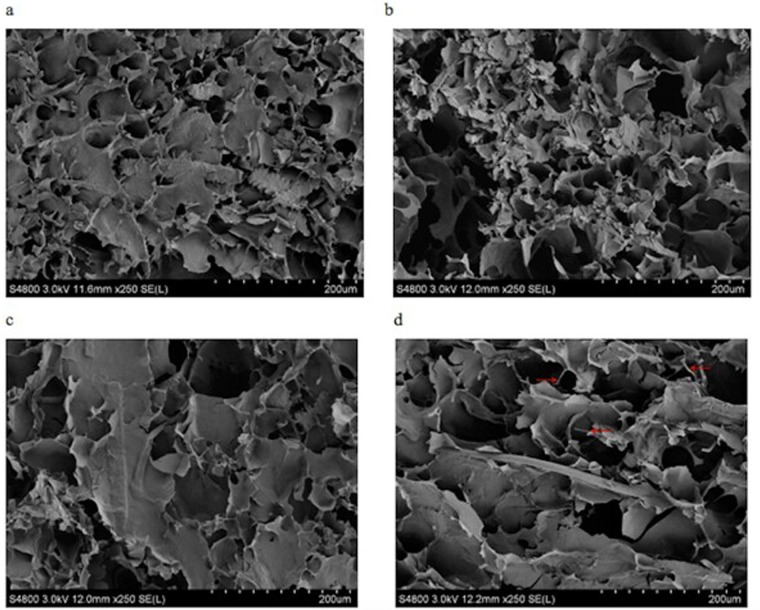
Scanning electron micrographs (SEM) of gelatinized wheat starch gel with 10, 20, 30, 40% 180-mesh wheat fiber.

Native wheat starch granules had a spherical structure [1], while many small pores were observed in the gelatinized wheat starch gel, and its dense structure was similar to a honeycomb construction (Fig 1). The SEM revealed the influence of the 10, 20, 30 and 40%, 60 mesh wheat fiber, and the 180 mesh wheat fiber on the gelatinized wheat starch gel (Figs 2 and 3). Because the SEM of mung bean starch and wheat starch with the wheat fiber were similar, only the gelatinized wheat starch gel was analyzed.

As can be seen from Fig 2a–2d and Fig 3a–3d, with increasing wheat fiber, the dense honeycomb structure of the starch gel gradually disappeared. The number of internal pores decreased, and a large lamellar structure was formed, with some filaments as well. One possible reason for this was that the wheat fibers existed in the form of small fragments [25]. There was a better compatibility between the small particles of wheat fiber and the starches, so the small particles participated more easily in the formation of the gel network structure. However, when the wheat fiber had a large particle size, the compatibility with the starch lessened. It was difficult for the fibers to participate in the formation of the gel network structure. There were larger and larger wheat fiber fragments which could be observed under the SEM.

When adding the 40% wheat fiber, the rod-shaped wheat fiber could be seen in the picture (Figs 2d and 3d). Some of the wheat fiber filaments were interspersed in the gel network structure of the starch, while some of them combined with the starches. One reason may be that the starches belonged to smaller molecules when compared with the wheat fiber. When the added amount of wheat fiber reached a maximum of 40%, the starches, as small molecules, were attached to the macromolecules of the wheat fiber and formed the large lamellar structure.

Compared with the gelatinized wheat starch gel with 180 mesh wheat fiber, the gelatinized wheat starch gel with the 60 mesh wheat fiber could form a stronger and more solid structure, with less small fragments attached to the surface. This may be because the structure of the 180 mesh wheat fiber was broken down extensively and produced more irregular, smaller fragments. This result coincided with the results of the TPA (Table 2). Alvarez et al. and Lovedeep et al. studied the microstructure of sisal fiber/starch-based composites, and starch—cassia gum mixtures, respectively. Their findings were similar to ours [29, 30].

## Conclusions

In the presence of wheat fiber with different added percentages and different particle sizes, the PVs of both starches were increased about two times, while the PTs were lessened, when compared to the control. Starches had the highest PVs with the 40%, 60 mesh wheat fiber. The TPA data of the blends showed higher hardness and lower cohesiveness when compared to the control. The starches with 40%, 60 mesh wheat fiber showed the highest hardnesses of 147.78 and 1032.11g, respectively. These values were about two times larger than those of the control. The SEM of the wheat starch-wheat fiber gel showed that with increasing wheat fiber, the dense honeycomb structure of the starch gel gradually diminished, and the internal pores lessened. A large lamellar structure was formed with some filamentous connections as well. Our research will be helpful for the development of functional wheat starch and mung bean starch products (starch noodles, vermicelli, sheet jelly, etc.) with enriched wheat fiber.
